# Apathy Classification Based on Doppler Radar Image for the Elderly Person

**DOI:** 10.3389/fbioe.2020.553847

**Published:** 2020-11-03

**Authors:** Naoto Nojiri, Zelin Meng, Kenshi Saho, Yucong Duan, Kazuki Uemura, C. V. Aravinda, G. Amar Prabhu, Hiromitsu Shimakawa, Lin Meng

**Affiliations:** ^1^College of Information Science and Engineering, Ritsumeikan University, Kusatsu, Japan; ^2^College of Science and Engineering, Ritsumeikan University, Kusatsu, Japan; ^3^Faculty of Engineering, Toyama Prefectural University, Imizu, Japan; ^4^Data Science and Technology Department, Hainan University, Haikou, China; ^5^Department of Computer Science and Engineering, NMAM Institute of Technology, NITTE, Karkala, India

**Keywords:** apathy classification, doppler radar image, the elderly person, machine learning, deep learning

## Abstract

Apathy is a disease characterized by diminished motivation not attributable to a diminished level of consciousness, cognitive impairment, or emotional distress. It is a serious problem facing the elderly in today's society. The diagnosis of apathy needs to be done at a clinic, which is particularly inconvenient and difficult for elderly patients. In this work, we examine the possibility of using doppler radar imaging for the classification of apathy in the elderly. We recruited 178 elderly participants to help create a dataset by having them fill out a questionnaire and submit to doppler radar imaging while performing a walking action. We selected walking because it is one of the most common actions in daily life and potentially contains a variety of useful health information. We used radar imaging rather than an RGB camera due to the greater privacy protection it affords. Seven machine learning models, including our proposed one, which uses a neural network, were applied to apathy classification using the walking doppler radar images of the elderly. Before classification, we perform a simple image pre-processing for feature extraction. This pre-processing separates every walking doppler radar image into four parts on the vertical and horizontal axes and the number of feature points is then counted in every separated part after binarization to create eight features. In this binarization, the optimized threshold is obtained by experimentally sliding the threshold. We found that our proposed neural network achieved an accuracy of more than 75% in apathy classification. This accuracy is not as high as that of other object classification methods in current use, but as an initial research in this area, it demonstrates the potential of apathy classification using doppler radar images for the elderly. We will examine ways of increasing the accuracy in future work.

## 1. Introduction

Apathy is a disease characterized by diminished motivation not attributable to a diminished level of consciousness, cognitive impairment, or emotional distress (Marin, 1990, 1991; Marin et al., 1991). It has a relationship with others diseases such as Parkinson's, Alzheimer's, and stroke, all of which tend to befall elderly people and threaten their health and well-being (Landes et al., 2001; Fuh et al., 2005; Caeiro et al., 2013; Pagonabarraga et al., 2015). Studies have shown that roughly 47% of patients with Alzheimer's disease also suffer from apathy (Fuh et al., 2005). However, to get an apathy diagnosis, elderly patients need to go to a clinic, which is both inconvenient for them and sometimes physically difficult. A computer vision system for assistance with apathy diagnosis in remote operation has been developed (Happy et al., 2019), but since it uses images of the patient's face, problems related to privacy protection arise. Another issue is that patients typically need to exhibit subjective symptoms before seeking a doctor, but apathy rarely has subjective symptoms, particularly among the elderly who often live in solitude. Hence, elderly people may delay getting diagnoses and miss out on the best treatment period.

Society is currently facing a rapid increase in the aging population—especially in Japan, where the percentage of the population aged 65 and over (elderly) is 28.1%. As of 2018, the population aged between 65 and 74 years was 13.9% and aged 75 years and over was 14.2%. By 2065, these numbers are expected to increase to 38.4% for ages 65+ and 25.6% for ages 75+ (CabinetOfficeJapan, 2019). Hence, developing a more convenient apathy assessment is becoming an important issue.

In this work, we examine the use of Doppler radar imaging for the classification of apathy in the elderly. Our objective is to encourage earlier access to apathy assessment. Doppler radar imaging is advantageous because it does not use face images, which helps with protecting privacy, and the equipment it uses is simple enough to set up that apathy checks can be performed routinely without any special preparation. Besides, because the Doppler radar directly measures the velocities, the accuracy of velocity measurement is better than other optical sensor techniques that mainly measures position information (Li et al., 2019). Furthermore, with its applicability to low-light conditions and to persons wearing ordinary clothes as its advantages, the Doppler radar has been investigated for using in home and hospital health monitoring applications in recent years (Seifert et al., 2019).

Unfortunately, as little research has been done in this area, it is not clear which action is best suited for apathy classification. Hence, we select one of the most normal actions in daily life: walking. Walking has a deep relationship with health condition and has been used since 1984 for clinical gait assessment in the neurologically impaired (Holden et al., 1984). It is easy to see how the action of walking relates to health condition; for example, stroke victims often have difficulty controlling their body when walking. Recently, researchers found that the action of walking can reveal a lot of a person's health information, including age (Handri et al., 2009; Makihara et al., 2011) and chronic illness (Pitta et al., 2005; Jehn et al., 2009; Rabinovich et al., 2013).

In this study, we created the Elderly Person Apathy Doppler Radar Image Dataset (EPADRI Dataset) with the help of elderly people aged 65 years or more. We had each participant fill out a questionnaire to determine if they had apathy or non-apathy and then perform a walking action under doppler radar to obtain experimental images. We then combined image processing and machine learning to perform apathy classification using the EPADRI Dataset.

As pre-processing, we utilize a simple image processing for extracting the features from the radar images. In this processing, a walking doppler radar image is separated into four parts by the vertical and horizontal axes and then binarization is applied to count the features of the eight parts for training and classification by machine learning. We apply four patterns for binarization—red channel, green channel, blue channel, and YUV—and slide the threshold of binarization from 50 to 220 to determine the optimized value. Finally, the number of feature points is used for the apathy classification.

As we know, machine learning models and Numerical Analysis methods are widely used in the Biology and Bioinformatics (Lu et al., 2015, 2019; Saho et al., 2020). In this work, we applied seven machine-learning models to a classification task: a support vector machine (SVM) (Vapnik, 1998), K-nearest neighbor (KNN) (Naomi, 1992), naive Bayes, decision tree (Quinlan, 1886), random forest (Breiman, 2001), an ensemble model (Opitz and Maclin, 1999), and our proposed neural network model (Homma et al., 1998).

This is the first paper to tackle apathy classification by using doppler radar images of walking action for the elderly. Our experimental results demonstrate the effectiveness of this approach.

The contributions of this work are as follows.

- We constructed the Elderly Person Apathy Doppler Radar Image Dataset (EPADRI Dataset), which is the first dataset for apathy classification of the elderly by walking action.
- We demonstrate the effectiveness of using doppler radar images of walking actions for apathy classification and show that it both ensures privacy protection and is convenient to use.
- We propose image processing and machine learning for apathy classification of the elderly and describe the optimized threshold of binarization, color channel, and machine learning models.

Section 2 discusses related work on apathy classification and health care research on walking and doppler radar imaging. We present our dataset in Section 3. Section 4 introduces our approach, featuring the machine learning used in the experiments. The experimentation results on the apathy classification task are shown in section 5. Section 6 discusses the contributions of this work as well as the limitations. We conclude in section 7 with a brief summary and mention of future work.

## 2. Related Work

### 2.1. Research on Apathy Classification

Apathy, which is derived from the Greek pathos, or passion, is conventionally defined as the absence or lack of feeling, emotions, interest, or concern (Marin, 1990). Robert et al. define apathy in clinical terms as including diminished motivation not attributable to a diminished level of consciousness, cognitive impairment, or emotional distress (Marin, 1990, 1991). Apathy occurs in several neurological and psychiatric disorders and seems to have a relationship with Parkinson's disease, Alzheimer's disease, stroke, etc., which often appear in the elderly (Landes et al., 2001; Caeiro et al., 2013; Pagonabarraga et al., 2015). Hence, the assessment and early diagnosis of apathy is quite important, especially among the elderly.

Currently, patients need to go to a clinic for an apathy diagnosis, which usually entails medical personnel administering time-consuming clinical interviews and questionnaires. Such interviews, and getting to the clinic itself, are sometimes inconvenient and can be very hard on the elderly. This is unfortunate because if diagnosis is delayed, an elderly person will miss out on the best treatment period. Several researchers have examined the use of computer science-based methods such as computer vision and machine learning for apathy classification. Happy et al. classified apathetic and non-apathetic patients by machine learning in which the analysis target was facial dynamics entailing both emotion and facial movement (Happy et al., 2019). They administered apathy assessment interviews to 45 participants, which included short video clips with wide face pose variations, very low-intensity expressions, and insignificant inter-class variations, and reported the accuracy of 84%.

Liu et al. designed a system called ECOCAPTURE that assesses apathy in a quantitative and objective manner. It consists of observation of a patient's behavior in a multi-step scenario reproducing a brief, real-life situation by using a single 3D accelerometer under an ecological condition. An evaluation with 30 patients and 30 healthy individuals showed that ECOCAPTURE is a promising technique for more precise assessment of apathy (Liu et al., 2018).

### 2.2. Research on Walking in the Filed of Healthcare

Walking is one of the most common actions in daily life and can reveal abundant health information such as age and chronic illness. In 1984, walking ability was utilized for clinical gait assessment in the neurologically impaired (Holden et al., 1984). It is easy to see that the action of walking has some relationship with health condition; for example, stroke victims often have difficulty controlling their body when walking. More recently, researchers have found that measuring a patient's ability to walk is important in the diagnosis of chronic illness (Pitta et al., 2005; Jehn et al., 2009; Rabinovich et al., 2013). This has led to research into devices that protect patients by monitoring walking, such as a natural walking monitor for pulmonary patients used in conjunction with a mobile phone (Juen et al., 2015).

Researchers have also found that the walking action can be linked to an individual's age (Handri et al., 2009; Makihara et al., 2011). This has led to the development of devices like the walking-age analyzer for healthcare applications (Jin et al., 2014).

### 2.3. Research on Doppler Radar Imaging in Health Care Industry

Doppler radar imaging is a promising method in the e-health industry due to its assurance of privacy protection and the fact that it is non-wearable. Li et al. designed e-health applications by using passive doppler radar as a non-contact sensing method to capture human body movements, recognize respiration, and measure physical activities. Techniques related to health monitoring include micro doppler extraction for breathing detection and a support vector machine classifier utilized for physical activity recognition. Non-contact passive doppler radar has proven to be a complementary technology to meet the challenges of future healthcare applications (Li et al., 2018).

Chen et al. also applied radar imaging for classifying the six key activities of interest in the e-health area and found that it is effective for activity recognition (Chen et al., 2016).

Our motivation in the present study is to use radar images of walking action for apathy classification in the elderly. Our approach circumvents the issues in previous research because walking action is a normal daily action, which makes it simple to assess, and radar imaging protects privacy and is non-wearable. As such, we hope to make apathy assessment for the elderly simpler and more convenient. In this work, we examined seven machine-learning models for classification and a simple image processing method for feature extraction.

## 3. Creation of the Elderly Person Apathy Doppler Radar Image Dataset (EPADRI Dataset)

We created the Elderly Person Apathy Doppler Radar Image Dataset (EPADRI Dataset) for training the machine-learning model and testing the accuracy of apathy classification.

We recruited 178 elderly people to help create the EPADRI Dataset. These individuals had previously filled out a Japanese version of a questionnaire known as Apathy Scale (Starkstein et al., 1992; Okada et al., 1997) we administered for apathy classification. The Apathy Scale is one of the generally used test to classify the Apathy in the field of physiotherapy and epidemiology and its effectiveness is validated in numerous studies (den Brok et al., 2015). Of the participants, 81 were between 65 and 75 years old and 98 were between 76 and 94 years old. All participants were Japanese and the questionnaire and answers were in Japanese.

The experimental protocol was approved by the local ethics committee (Toyama Prefectural University, approval no. H29-1). Participants were provided with written and verbal instructions of the testing procedures, and written consent was obtained from each participant prior to testing.

### 3.1. Questionnaire for Apathy Classification

Table 1 lists the apathy questionnaire items in the Apathy Scale (Starkstein et al., 1992). Table 2 lists the responses and points. Points were tallied to judge the apathy situation. Participants with a score of 16 or more were judged to be apathetic people for the Japanese version of the Apathy Scale as verified in Okada et al. (1997).

**Table 1:** Questionnaire items.

	**Questions**
Q1	Do you want to study something new?
Q2	Do you have any interests?
Q3	Are you interested in your health?
Q4	Can you focus on things?
Q5	Do you always want to do something?
Q6	Do you have plans or goals for the future?
Q7	Are you willing to try doing something new?
Q8	Do you spend time doing something every day?
Q9	Does someone have to tell you to do something every day?
Q10	Are you indifferent to anything?
Q11	Is there anything that interests you?
Q12	Do you do nothing unless someone tells you?
Q13	Do you ever feel not happy, not sad, but somewhere in the middle?
Q14	Do you think you are motivated?

**Table 2:** Questionnaire responses and points.

**Selection**	**Points**
No	0
A little	1
Yes	2
Very	3

### 3.2. Doppler Radar Image Creation

**Figure 1:**
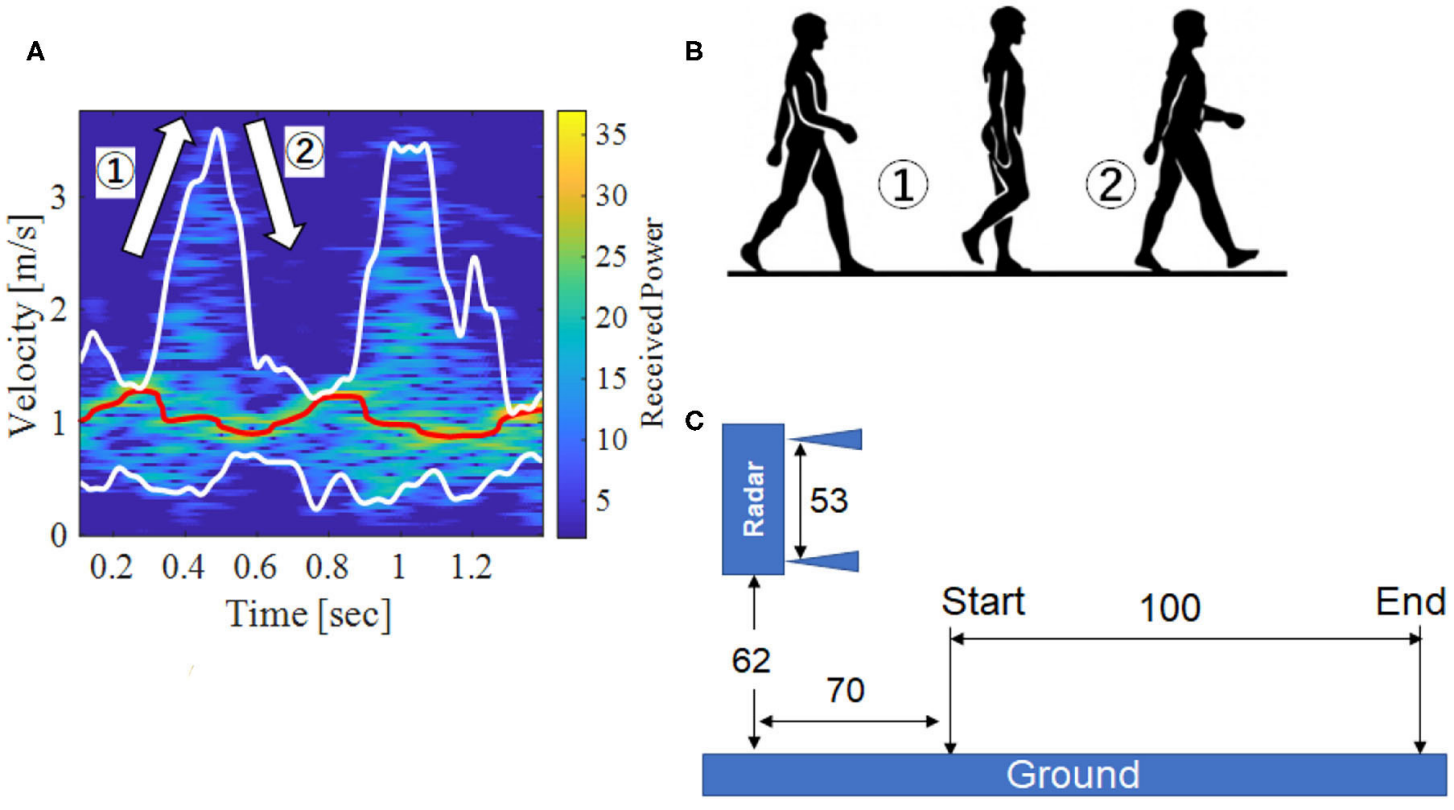
Doppler radar image and experimental environment. **(A)** Original image, **(B)** Walk process, **(C)** Experimental environment.

Figure 1 shows the creation of a radar image, where Figure 1A is an example of a doppler radar image with walking action and Figure 1B shows the walk process that is taken. Figure 1C shows the experimental environment, where the radar size is about 53 cm, the height is 62 cm, the start point is about 70 cm from the radar, and the walking distance is 100 cm.

## 4. Apathy Classification by Machine Learning

**Figure 2:**
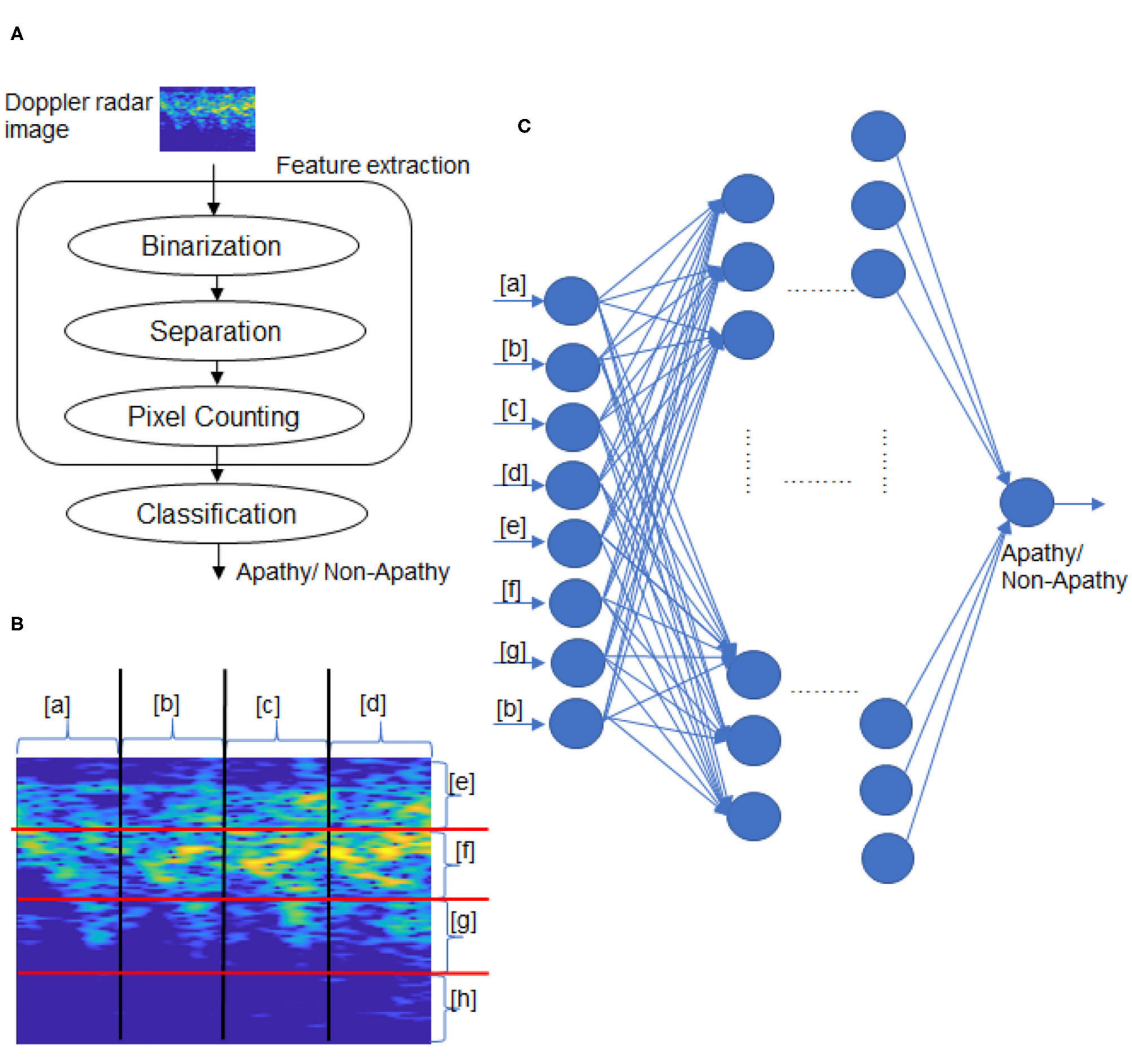
Overview of proposed method. **(A)** Classification flow, **(B)** Doppler radar image separation, **(C)** Classification by NN.

In this section, we propose our method for apathy classification that combines image processing with machine learning. Figure 2A shows the classification flow, which consists of feature extraction and classification.

We propose a simple image processing to extract the features from the walking radar image. Next, we apply seven machine-learning models, including an NN model we developed, to perform classification by using the extracted features.

Our objectives are two-fold. First, we want to demonstrate the possibility of performing apathy classification for the elderly by machine learning. Second, we want to determine the best model and best parameters by means of experimentation.

### 4.1. Feature Extraction

The feature extraction consists of binarization, image separation, and feature pixel counting, as shown in Figure 2A.

As discussed earlier, it is not clear which channel in an image is most suitable for apathy classification. We therefore focus on pixel configuration for the binarization and apply four kinds binarization: red channel, green channel, blue channel, and the Y of YUV. YUV is a color encoding system which encodes a color image taking human perception. The Y is defined as

$$\begin{array}{l} Y=0.299*r\left[i\right]\left[j\right]+0.587*g\left[i\right]\left[j\right]+0.114*b\left[i\right]\left[j\right] \end{array}$$

(Charles, 2003), where the r, g, and b is the red, green and blue channel, *i* and *j* describe the coordinates of pixel.

Threshold is a key parameter in binarization as it may influence the classification accuracy. In our threshold decision, when a pixel (*P*_*i, j*_) is more than the threshold, the pixel value is set to 255 (white pixel), and otherwise is set to 0. We set the pixel as one of four kinds (red channel, green channel, blue channel, and Y of YUV) and slide the threshold from 50 to 220 to determine the best value.

$$B_{i,j}=\left\{ \begin{array}{ll} 255 & \left(P_{i,j}>=threshold\right)\\ 0 & \left(otherwise\right) \end{array}\right.$$

After the binarization, every image is separated into four parts by the vertical and horizontal axes. An example of a separated image is shown in Figure 2, which includes lists items from *a* to *h*. The white pixel numbers of eight parts in the binarized image are counted. Finally, the eight numbers are decided as the features for apathy classification by the following machine-learning models.

### 4.2. Classification of Machine-Learning Models

This subsection introduces the seven machine-learning models we examined to determine which one was most suitable for apathy classification: a support vector machine (SVM) (Vapnik, 1998), k-nearest neighbor (KNN), naive Bayes, decision tree, random forest, an ensemble model, and our proposed neural network (NN).

#### 4.2.1. SVM

An SVM is a supervised learning model for the boundary decision and classification of data by maximum-margin hyperplane. The most basic idea is classification using linear separability. Data are defined as *Data* = {*X, Y*}, where *X* = {*X*_1_, …*X*_*N*_} is the feature of the input data and *Y* = {*y*_1_, …*y*_*N*_} is the class label of each input data. The boundary decision is defined as *w*^*T*^*X* + *b* = 0, where *w* is the normal vector to a hyperplane and *b* is the intercept. The constraint condition is $y_{i}\left(w^{t}X_{i}+b\right)>=1$, which is used for the boundary decision and classification.

#### 4.2.2. KNN

k-nearest neighbor is a basic classifier that calculates the k closest training in the feature space (Naomi, 1992). Data are usually defined as *X* = {*x*_1_, …, *x*_*N*_} and *Y* = {*y*_1_, …, *y*_*N*_}. Absolute distance measuring, Euclidean distance measuring, or some other distance function is used for calculating the minimum distance. In this study, we define two k: one for the classification of apathy and the other for non-apathy.

#### 4.2.3. Naive Bayes

Naive Bayes is a simple technique for constructing classifiers. In abstract terms, naive Bayes is a conditional probability model: when given a problem instance to be classified, represented by a vector *x* = {*x*_1_, …, *x*_*n*_} representing some *n* features, it assigns to this instance probabilities *p*(*C*_*k*_∣*x*_1_, …, *x*_*n*_) for each of *K* possible outcomes or classes *C*_*k*_.

The problem with the above formulation is that if the number of features n is large or if a feature can take on a large number of values, basing such a model on probability tables is infeasible. We therefore reformulate the model to make it more tractable. Using Bayes' theorem, the conditional probability can be decomposed as

$$\begin{array}{l} p\left(C_{k}|x\right)=p\left(C_{k}\right)p\left(x|C^{,}\right)÷P\left(x\right). \end{array}$$

It can also be

$$\begin{array}{l} posterior=prior\times likelihood÷evidence. \end{array}$$

#### 4.2.4. Decision Tree

Decision tree is a decision support tool that uses a tree-like model of decisions and their possible consequences, including chance event outcomes, resource costs, and utility. It is typically used to display an algorithm that only contains conditional control statements (Quinlan, 1886).

In the decision tree (two-class) model, the correct decision tree for class *Data* = *X, Y*, number of classes *P* is *p*, and the number of the another class *N* is *n*. Any correct decision tree for *Data* will classify objects in the same proportion as their representation in *Data*. An arbitrary object will be determined to belong to the class *P* with probability *p*(*p* + *n*) and the class *N* with probability *n*÷(*p* + *n*).

To classify an object, the expected information is generated by

$$\begin{array}{l} I\left(p,n\right)=-p÷\left(p+N\right)log_{2}p÷\left(p+n\right)\\ \;-n÷\left(p+n\right)log_{2}n÷\left(p+n\right). \end{array}$$

The expected information required for the tree with A as a root is then obtained as the weighted average

$$\begin{array}{l} E\left(A\right)=\overset{v}{\underset{i=1}{\sum }}\left(P_{i}+n_{i}\right)÷\left(p-n\right)I\left(P_{i},n_{i}\right), \end{array}$$

where the weight for the *i*th branch is the proportion of the objects in C that belong to *X*_*i*_. The information gained by branching on A is therefore *gain*(*A*) = *I*(*p, n*) − *E*(*A*).

#### 4.2.5. Random Forest

Random forest is a combination of tree predictors in which each tree depends on the values of a random vector sampled independently and where all trees in the forest have the same distribution (Breiman, 2001).

The point is to create a group of decision trees with low correlation by using randomly sampled training data and randomly selected explanatory variables.

First, *m* training sets are generated by a bootstrap model. Then, for each training set, a decision tree is constructed. When a node searches for a feature and splits it, this is not to find the feature that can maximize the index (such as information gain) but to randomly extract various features and find the optimal solution among them, which is then applied to the node and split again. The random forest model uses the idea of bagging, that is, integrating, so is actually equivalent to sampling samples and features, which means it can avoid overfitting. The prediction stage includes the bagging strategy, classified voting, and regression of mean value.

#### 4.2.6. Neural Network (NN)

An NN is a mathematical model that mimics the network structure of nerve cells (neurons) in the brain (Cun, 1989; Homma et al., 1998). It builds multiple layers of interconnected nodes for training data and is typically used for pattern recognition, data classification, and future prediction.

In this work, we propose an NN model that consists of five layers, as follows.

- Input layer: 8-node, activation is relu.
- Second layer: 16-node, activation is relu.
- Third layer: 32-node, activation is relu.
- Fourth layer: 64-node, activation is relu.
- Output layer: 1-output, activation is sigmoid

The training epoch is set to 50. The confidence is set to 0.5, which means when the confidence of the apathy classification is >0.5, the prediction result is judged as apathy, and otherwise as non-apathy.

#### 4.2.7. Ensemble Model

The ensemble model performs predictions by means of a combination of several basic prediction models. The key idea is to generate a final prediction result based on the principle of majority voting with respect to the prediction results of all the models (Opitz and Maclin, 1999; Polikar, 2006).

In this method, SVM, random forest, NN, and KNN are used as the basic models. *apVote* and *noapVote* are calculated by counting the number of apathy and non-apathy prediction results, respectively. The final result is then predicted by

$$Finalresult=\left\{ \begin{array}{ll} Apathy & \left(apVote>=noapVote\right)\\ NonaApathy & \left(otherwise\right) \end{array}\right.$$

## 5. Experimentation

### 5.1. Experimental Conditions

We used 178 walking radar images of 178 elderly participants in our experiment. A total of 150 images were used for training (48 apathy, 102 non-apathy), and the remaining 28 images were used for testing (eight apathy, 20 non-apathy). Each participant had one doppler radar image of a walking action.

Python 3.7 was used for programming the feature exaction and machine learning design. Anaconda was used as the standard platform. The hardware environment was a CPU (core i7 8th Gen, memory: 32 GB).

### 5.2. Overview of Accuracy

**Figure 3:**
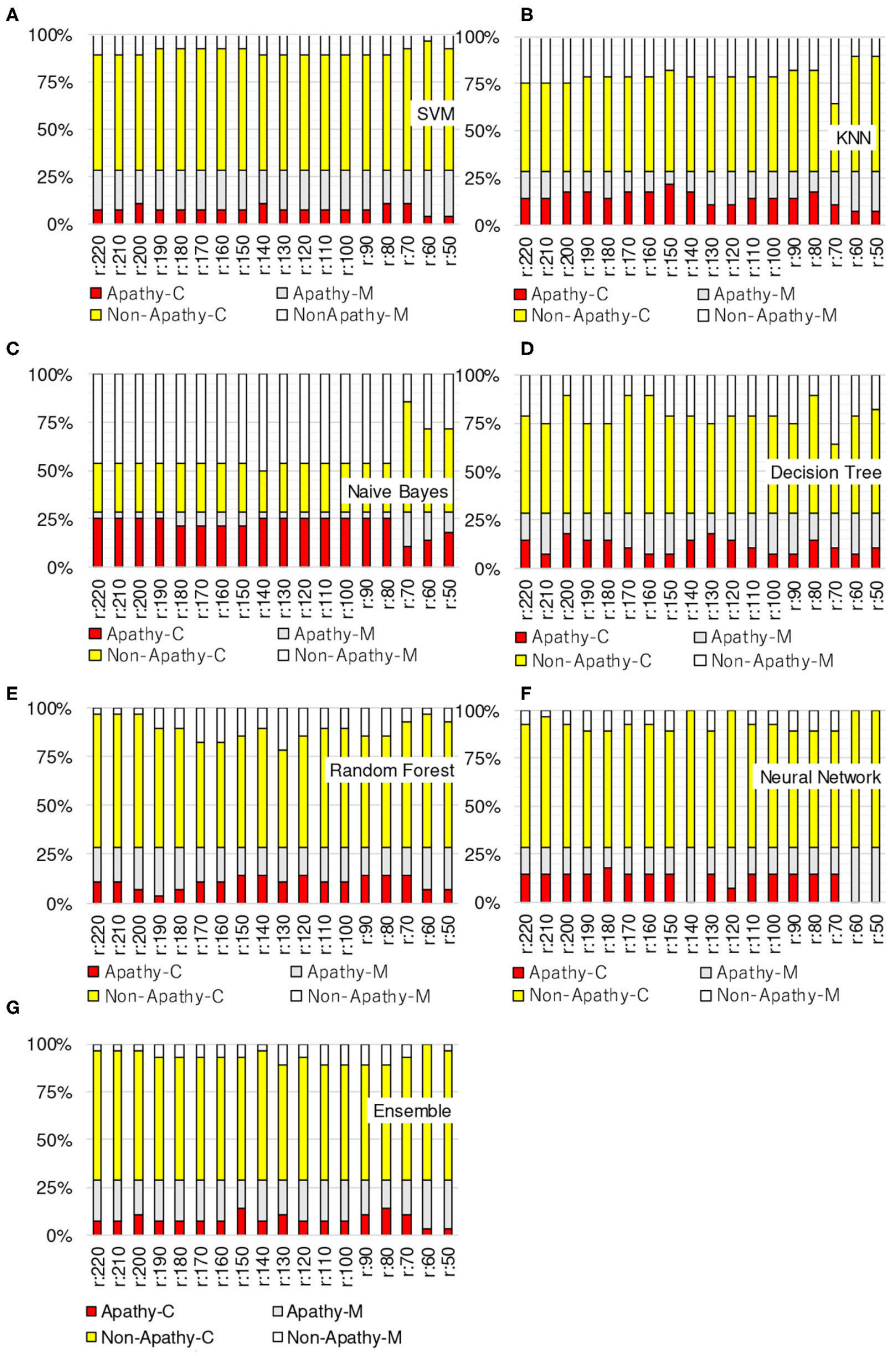
Experimental results: using red channel. **(A)** SVM, **(B)** KNN, **(C)** Naive Bayes, **(D)** decision tree, **(E)** random forest, **(F)** neural network, **(G)** ensemble.

**Figure 4:**
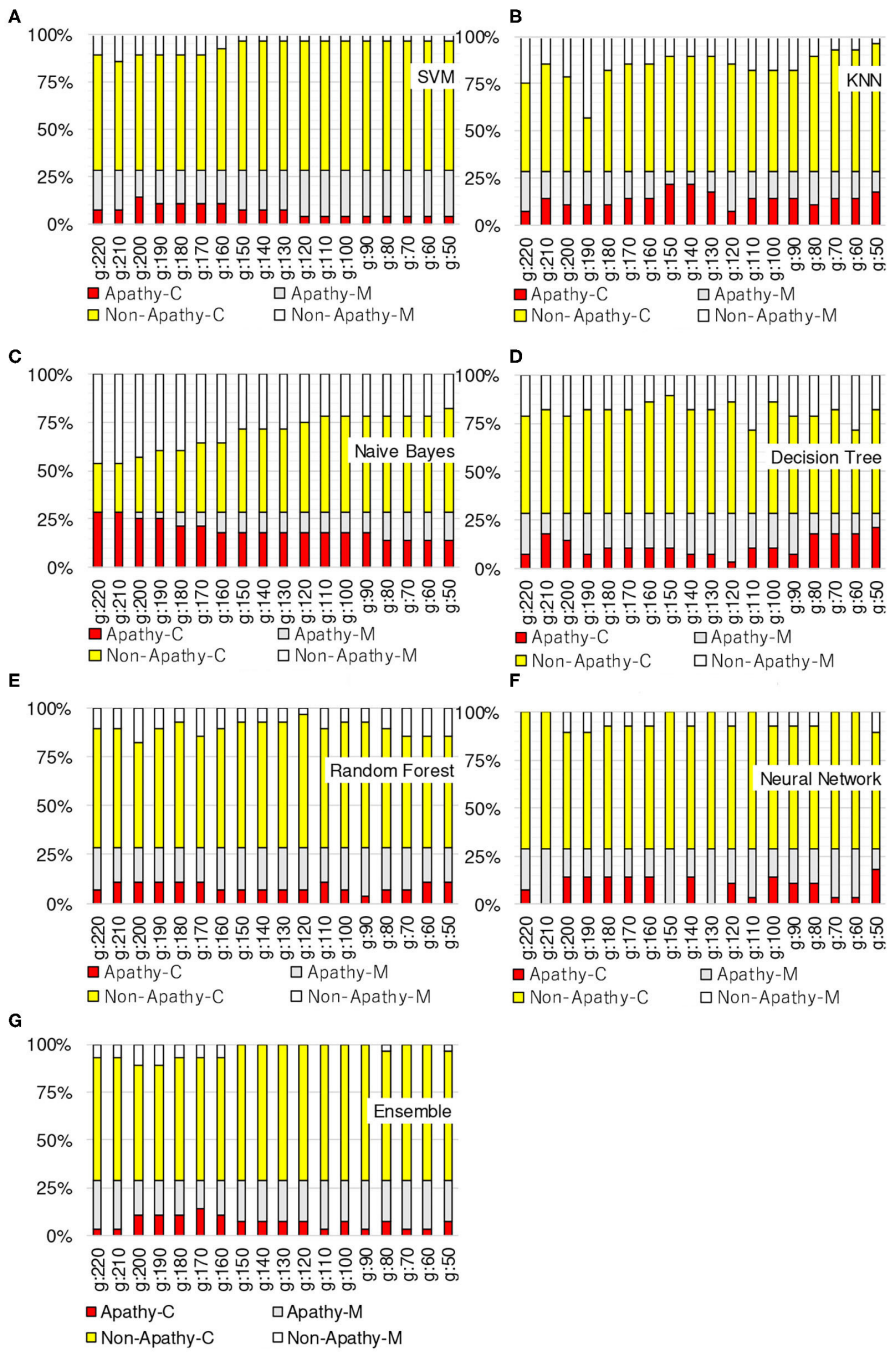
Experimental results: using green channel. **(A)** SVM, **(B)** KNN, **(C)** Naive Bayes, **(D)** decision tree, **(E)** random forest, **(F)** neural network, **(G)** ensemble.

**Figure 5:**
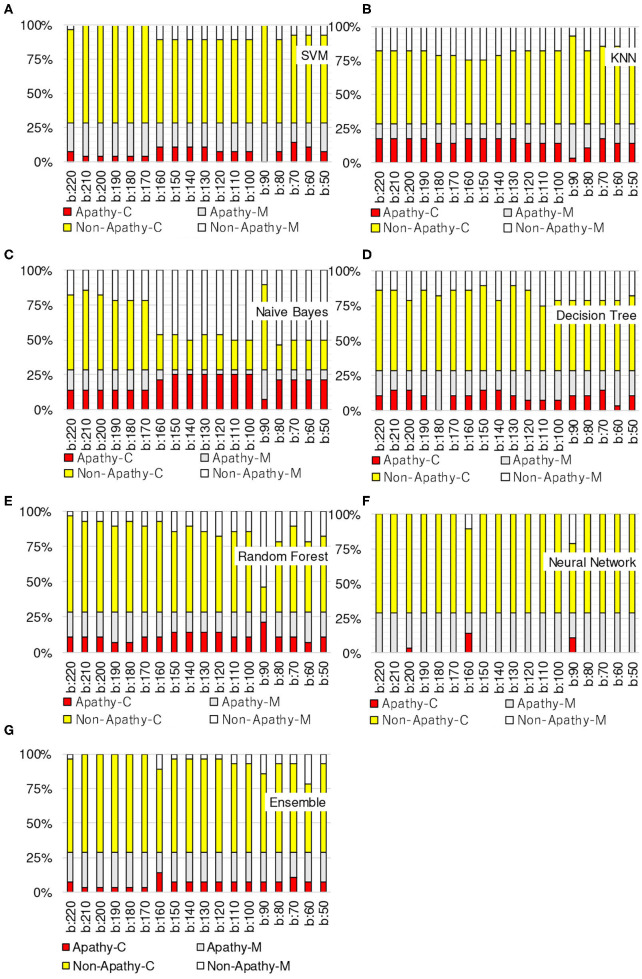
Experimental results: using blue channel. **(A)** SVM, **(B)** KNN, **(C)** Naive Bayes, **(D)** decision tree, **(E)** random forest, **(F)** neural network, **(G)** ensemble.

**Figure 6:**
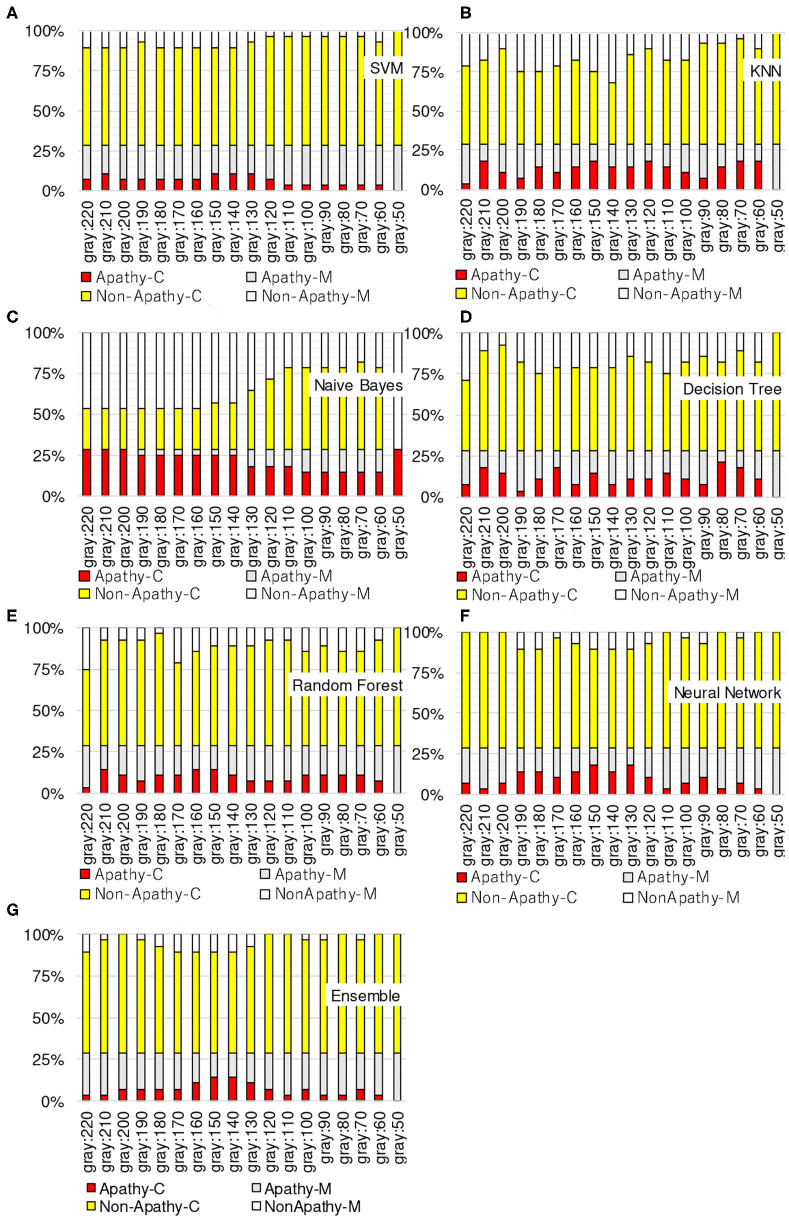
Experimental results: using Y of YUV. **(A)** SVM, **(B)** KNN, **(C)** Naive Bayes, **(D)** decision tree, **(E)** random forest, **(F)** neural network, **(G)** ensemble.

Figures 3–6 show the apathy classification accuracy when using only red channel, blue channel, green channel, and Y of YUV, respectively.

In these figures, the horizontal axis shows the threshold of binarization and the vertical axis shows the accuracy. Apathy-C denotes the correct classification rate of Apathy, Apathy-M the incorrect classification rate of apathy, Non-Apathy-C the correct classification rate of Non-Apathy, and Non-Apathy-M the incorrect classification rate of Non-Apathy. We should point out that this dataset was somewhat limited, as about 28.5% of the testing data was apathy data.

The seven sub-figures in each figure depict the respective apathy classification accuracy of each of the seven machine-learning models.

#### 5.2.1. Red Channel

Figure 3 shows the apathy classification accuracy of using only the red channel. We found that SVM, decision tree, random forest, and ensemble performed poorly in Apathy-C. Naive Bayes achieved a good accuracy in Apathy-C, but its accuracy in Non-Apathy-C was very low. On the other hand, Naive Bayes achieved slight improvement in Non-Apathy-C during the threshold is 70 to 50. However, accuracy in Apathy-C is very low in these thresholds. Hence, its total accuracy (Non-Apathy-C + Apathy-C) was low.

In KNN, the total accuracy (Non-Apathy-C + Apathy-C) was more than 64% in the case of the threshold from 140 to 220, and in NN, the total accuracy was more than 75% in the case of the threshold from 150 to 220.

#### 5.2.2. Green Channel

Figure 4 shows the apathy classification accuracy of using only the green channel. As with the experiment using the red channel, SVM, decision tree, random forest, and ensemble performed poorly in Apathy-C, and naive Bayes performed poorly in Non-Apathy-C.

In contrast to the results for the red channel, here KNN had a total accuracy (Non-Apathy-C + Apathy-C) of more than 65% in the case of the threshold from 130 to 170, and from 50 to 70. NN achieved a total accuracy of more than 75% in the case of the threshold from 160 to 200.

#### 5.2.3. Blue Channel

Figure 5 shows the apathy classification accuracy of using only the blue channel. The same as when using the red and green channels, SVM, decision tree, random forest, and ensemble performed poorly in Apathy-C, and naive Bayes performed poorly in Non-Apathy-C.

NN also performed poorly here, and missed almost all of the apathy images. KNN did not achieve an accuracy of more than 71% in total. These results demonstrate that using only the blue channel degrades the accuracy.

#### 5.2.4. Y of YUV

Figure 6 shows the apathy classification accuracy of using only the Y of YUV. As with the experiments with the red, green, and blue channels, SVM, decision tree, random forest, and ensemble performed poorly in Apathy-C, and naive Bayes performed poorly in Non-Apathy-C.

In addition, as in the experiment with the blue channel, KNN did not achieve an accuracy of more than 71% in total. As for NN, the total accuracy was more than 75% in the case of the threshold from 150 to 190.

### 5.3. Conclusion on Experimental Results

The results of the above experiments demonstrate that SVM, decision tree, random forest, and ensemble are not appropriate for use as machine-learning models for apathy classification of the elderly using doppler radar imaging. We conclude that KNN and NN are better models.

In terms of color channel, we found that the blue channel is not effective. Also, the Y of YUV is no better than the red or green channels, as Y is calculated using the blue channel. The accuracy of using Y is also just as bad as when using the blue channel, as only the slightest coefficient (0.114) is used for calculating Y.

When comparing all of the models and all of the thresholds, the proposed NN performed the best, with a total accuracy of more than 75%. The optimal threshold is from 150 to 190 when using red channel, green channel, and Y of YUV.

**Figure 7:**
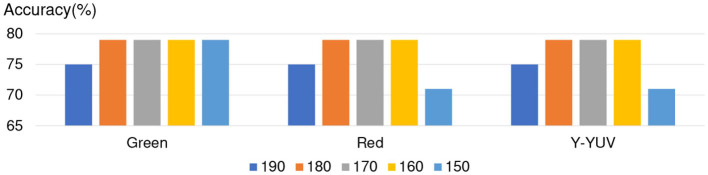
Channels discussion about NN.

For giving more accurate analysis about NN, we list the experimental results about the accuracy of red channel, green channel, and Y of YUV during the threshold from 150 to 190 in Figure 7. The experimental results show the three channels achieve the same accuracy in NN, especially in the threshold from 160 to 180. (Note: Almost all of the Apathy can not be recognized correctly in blue channel by NN which was shown in Figure 5.)

We performed additional experiments to see if we could further improve the performance of NN by changing the number of layers, activation functions, and epochs, but no improvements were observed. Hence, we consider the optimal model to be the proposed five-layer NN model (described in section 4).

**Figure 8:**
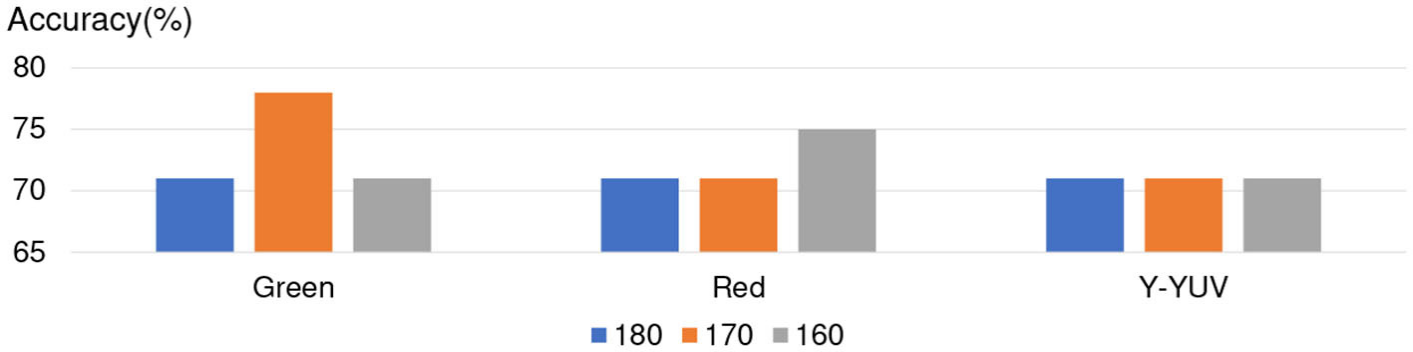
Discussion about Ensemble model.

Furthermore, for considering that Naive Bayes achieved better accuracy in Apathy-C and Neural Network achieved better accuracy in Non-Apathy-C. We only combines Naive Bayes and NN method in ensemble model for ensuring better accuracy. In term of the color channel and threshold, the red channel, green channel, and Y of YUV during the threshold from 150 to 190, are decided as the experimental condition. In these conditions, Naive Bayes achieved better accuracy in Apathy-C and Neural Network achieved better accuracy in Non-Apathy-C, by using the single model, respectively. The experimental results of Naive Bayes and NN combined ensemble model are listed in Figure 8, show that almost all of the cases only achieved 71% accuracy and were not better than NN.

**Figure 9:**
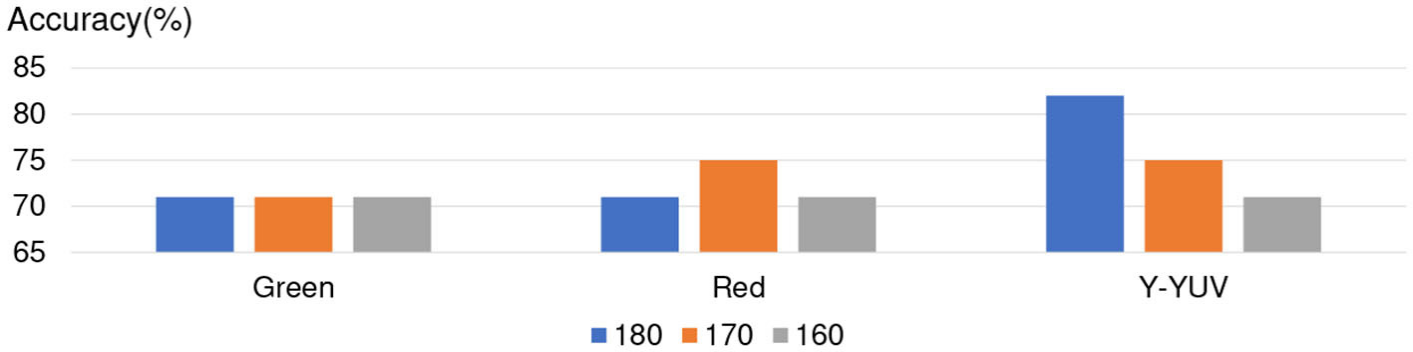
Discussion on experimental results of NN (validation = 0.2).

In conclusion, five-layer slight Neural Network achieved better apathy classification accuracy based on Doppler Radar Image by using the red channel, green channel, and Y of YUV during the threshold from 160 to 180. For proving credibility of the conclusion, we separated 20% of training data as the validation data, and trained the NN again. The experimental results are shown in Figure 9, and achieved the similar results as without validation data.

### 5.4. Optimization in Image Separation

The 4 × 4 image separation presented in Figure 2 considers the physical features of walking expressed on the radar images. Each separated image (Figure 2D) in [a]–[d] corresponds to the motion of each one step. The image [e] expresses the legs' motion in the stance phase of walking, [f] corresponds to body motion, and [g] expresses the legs' motion in the swing phase. The image [h] includes slight information on relatively large velocities of motions of toes or arms.

**Figure 10:**
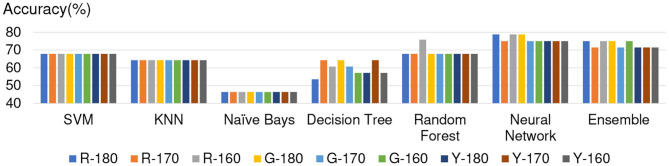
Experimental results of the 5 × 5 image separation.

**Figure 11:**
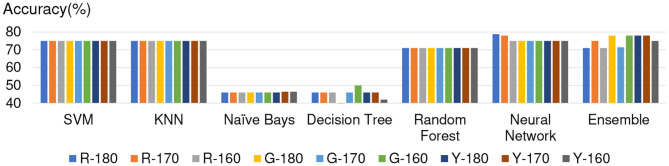
Experimental results of the 4 × 5 image separation.

For proving the optimization of 4 × 4 image separation, we also added two separated method experimentation, including 4 × 5 and 5 × 5. The results of accuracy of 5 × 5 is shown in Figure 10, and 4 × 5 is shown in Figure 11. The experimental results show that the additional experimentation can not achieve better accuracy than the 4 × 4 image separation.

Hence, the 4 × 4 image separation is an optimized method which can be proved by the characters of image and the experimentation results.

### 5.5. Experiment Using Deep-Learning Models

As various deep-learning models are proposed and used in the field of classification such as animal classification, characters classification etc. (Meng et al., 2018a,b, 2019), which achieved good accuracy. These models include LeNet (Lecun et al., 1998), AlexNet (Krizhevsky et al., 2012), GoogLeNet (Szegedy et al., 2015), VGG16, VGG19 (Simonyan and Zisserman, 2015), ResNet152V2 (He et al., 2015), Inception (Szegedy et al., 2016b), InceptionResNetV2 (Szegedy et al., 2016a), Xception (Chollet, 2017), and MobilNet (Howard et al., 2017) etc.

**Figure 12:**
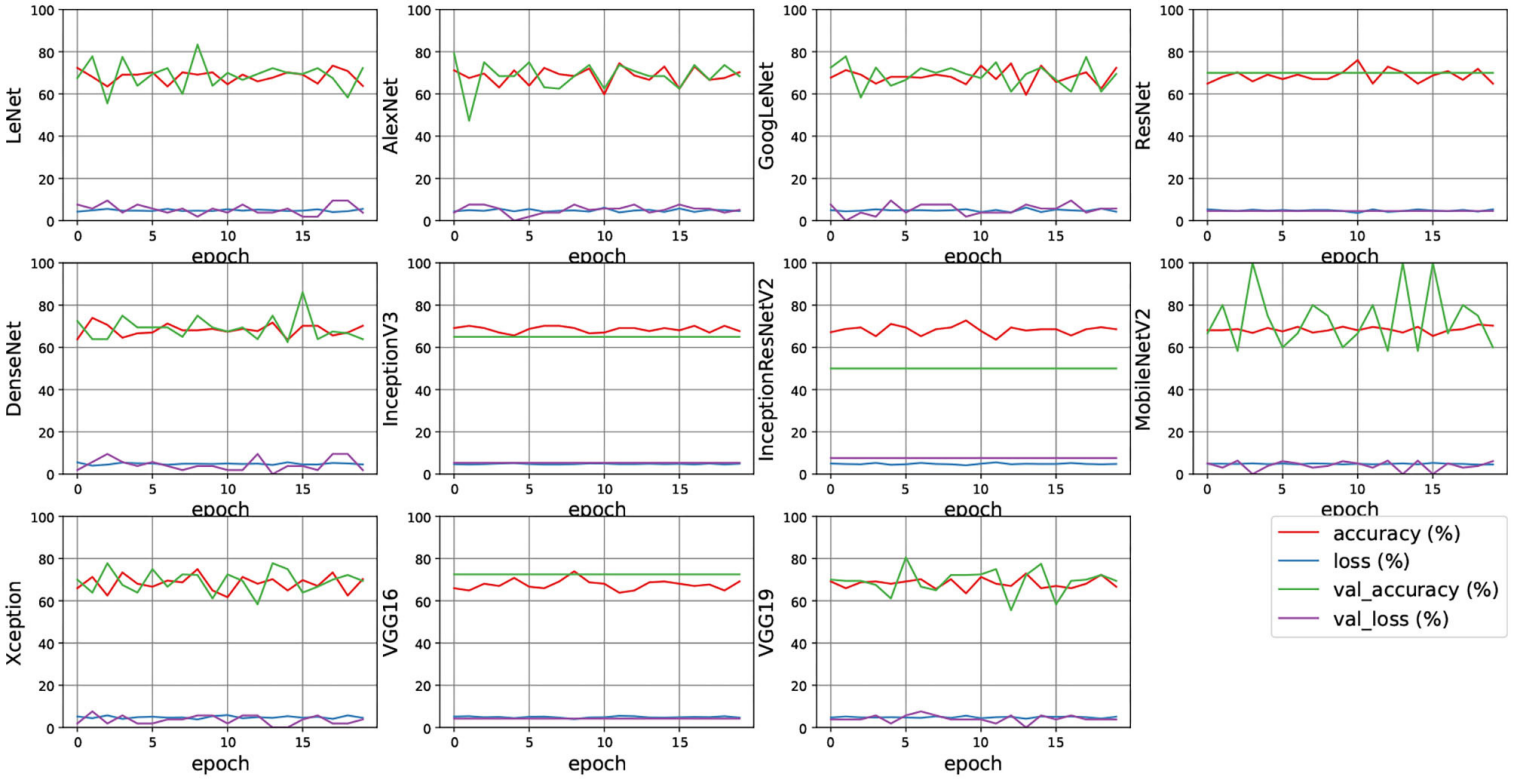
Experimental results of deep learning models.

We also applied these models for measuring the accuracy of Apathy classification. Figure 12 shows the experimental results of 11 state-of-the-art deep learning models for Apathy classification. The accuracy and the loss are listed. The results show that few of these models converged well such as ResNet, Inception, InceptionResNet, Mobile Net, VGG. Furthermore, the other models do not achieve better accuracy than the Machine learning models. Hence, the results demonstrate the difficulty of applying current deep-learning models to apathy classification using walking doppler radar images.

## 6. Discussion

### 6.1. Effectiveness and Significance of Research

In terms of feature extraction, we applied binarization using only the red channel, green channel, blue channel, and Y of YUV, and slid the threshold from 50 to 220. We found that all of the machine learning models could not achieve high accuracy when using only the blue channel. We discussed seven machine-learning models for apathy classification and showed that in the red channel, green channel, and Y of YUV, the threshold from 150 to 190 resulted in the accuracy of more than 75%. This result demonstrates the effectiveness and significance of this research.

We feel this accuracy should be improved in the future, but even so, our findings here demonstrate the possibility of achieving an apathy classification method for the elderly that is both convenient and protects their privacy.

### 6.2. Limitations

The limitation of this research is that the dataset is small, only 178 elderly person are participants help for creating the dataset. Even if it is very hard to realizing the current dataset, and the some current research only uses Dozens of participants such as paper (Happy et al., 2019) has 45 participants, and paper (Liu et al., 2018) has 30 patients. For improving the accuracy and realizing the Practical, the dataset set should be increased.

Another limitation is the set place of the drop radar and the walking action. As this is an initial study, we kept things simple by setting the drop radar in front of the participants and having them perform the walking action on command. For practical use in production and diagnosis, these limitations need to be considered.

## 7. Conclusion

In this paper, we have examined using a walking action doppler radar image for the classification of apathy in the elderly. Walking is a common action in daily life and radar imaging is a good method in terms of privacy protection, so using a walking action doppler radar image may help us to achieve a diagnostic method that is both convenient and protects privacy. For the apathy classification, we proposed a method that combines image processing with machine learning. We had 168 elderly people help create a dataset by filling out a questionnaire to determine if they exhibited apathy or non-apathy and then used the results to train and test seven machine-learning models. The image processing consists of binarization, image separation, and feature pixel counting to extract features. We focused on pixel configuration for the binarization and slid the threshold from 50 to 220 to determine the optimized value. We then applied seven machine-learning models including our proposed NN model to a classification task by using the extracted features. We found that, in the red channel, green channel, and Y of YUV, the threshold from 150 to 190 resulted in an accuracy of more than 75%. This demonstrates the effectiveness of our approach and suggests its potential for achieving an apathy classification method for the elderly that is both convenient and protects their privacy. Further the EPADRI Dataset and the classification code are opened in our Lab website for reproducible study [http://www.ihpc.se.ritsumei.ac.jp/Publication.html: Apathy Dataset and Classification Code(2020)]. In future work, we will improve the accuracy further by increasing the size of the dataset.

## Data Availability Statement

The raw data supporting the conclusions of this article will be made available by the authors, without undue reservation.

## Ethics Statement

The experimental protocol was approved by the local ethics committee (Toyama Prefectural University, approval no. H29-1). Participants were provided with written and verbal instructions of the testing procedures, and written consent was obtained from each participant prior to testing.

## Author Contributions

NN and ZM have the same contribution on experimentation and paper writing. KS and KU created the dataset and give advice on the data analysis. YD gave the advice on the data analysis on bio fields. CA and GA share the algorithms. HS and LM are supervisors of this project research. All authors contributed to the article and approved the submitted version.

## Conflict of Interest

The authors declare that the research was conducted in the absence of any commercial or financial relationships that could be construed as a potential conflict of interest.
